# The impact of a CD4 tiered service model on interlaboratory referral distances in South Africa

**DOI:** 10.4102/jphia.v16i1.1357

**Published:** 2025-09-23

**Authors:** Naseem Cassim, Manuel P. da Silva, Deborah K. Glencross, Lindi-Marie Coetzee, Wendy S. Stevens

**Affiliations:** 1 Wits Diagnostic Innovation Hub, Faculty of Health Sciences, University of the Witwatersrand, Johannesburg, South Africa; 2 National Priority Programme, National Health Laboratory Service, Johannesburg, South Africa; 3 Faculty of Health Sciences, University of the Witwatersrand, Johannesburg, South Africa

**Keywords:** CD4, distance, HIV, interlaboratory referrals, euclidean distance (ED)

## Abstract

**Background:**

South Africa has the world’s largest human immunodeficiency virus (HIV) pandemic. Most service gaps for cluster of differentiation 4 (CD4) testing were previously addressed.

**Aim:**

This study aimed to assess the impact of a tiered service on interlaboratory referral distances.

**Setting:**

Data are reported for CD4 testing that are referred from a national network of laboratories.

**Methods:**

Test volumes were extracted for source and testing laboratories from 2012 to 2021. The Euclidean distances (EDs) were calculated, with the annual and provincial medians reported and categorised (50 km, 51 km – 99 km, 100 km – 199 km, 200 km – 299 km and ≥ 300 km). The relationship between ED, referrals and turnaround time (TAT) was analysed. The change in the provincial median ED between 2012 and 2021 was calculated.

**Results:**

Data included 14 487 006 referrals. The median ED ranged from 55 km to 60 km. An ED category of 51 km – 99 km, 100 km – 199 km, 200 km – 299 km and ≥ 300 km was reported for 35.1%, 13.2%, 3.5% and 0.3% of the specimens. A negative linear correlation was reported for ED with referral volumes (–0.1540) and TAT (–0.2305). The provincial median ED ranged from 16 km (Gauteng) to 186 km (Northern Cape). Excluding the Northern Cape, a provincial ED of ≤ 100 km was reported. The percentage change in median ED between 2012 and 2021 ranged from –55.7% (Free State) to 0.8% (Mpumalanga). Two source laboratories reported a median ED > 300 km in 2021 (Springbok and Beaufort West).

**Conclusion:**

The study’s findings indicate that the decentralisation of services reduced the national median ED to below 60 km.

**Contribution:**

The tiered implementation improved accessibility, however, some coverage gaps still remain.

## Introduction

In 2000, the Actuarial Society of South Africa (ASSA) model reported that 400 000 people living with human immunodeficiency virus (HIV) would develop acquired immunodeficiency syndrome (AIDS) by 2003, with an estimated 1.4 million people requiring antiretroviral therapy (ART).^1,2^ To address the spread of the HIV pandemic, the South African government developed an operational plan to scale up access to ART, aiming to ensure that by 2009, all people living with HIV would have access to comprehensive care, management and treatment of HIV and AIDS (CCMT).^1,2^ The National Strategic Plan (NSP) of 2000 of South Africa provided a framework for interventions geared towards initiating and executing a comprehensive response to HIV, which included four key areas: (1) prevention, (2) treatment, care and support, (3) research, monitoring and surveillance and (4) legal and human rights.^3^ During this period, ART was not provided by public health facilities in South Africa.^1,4,5,6^

The purpose of the operational plan was to provide a comprehensive response to the HIV epidemic, focussed on care, management and treatment.^1^ The operational plan provided projections of the number of people living with HIV who would be eligible to receive ART.^1^ Budget estimates included factors such as survival and mortality of people on ART.^1,2^ For the financial periods between 2003–2004 and 2007–2008, an estimated forecast of R11.9 billion was required to scale up ART provision for 53 000 people living with HIV in 2003–2004, ramping up to 551 089 by 2008–2009.^1,2^ Based on these forecasts, South Africa commenced the provision of ART at public health facilities in April 2004.^4^

By 2023, South Africa had an estimated 7.7m people living with HIV, of which 5.9m were on ART.^7^ Local guidelines released in 2023 recommended clinical assessment and laboratory baseline investigations before the initiation of ART.^8^ However, people living with HIV can be initiated on ART without the laboratory results, provided they have no clinical evidence of tuberculosis (TB), meningitis or renal disease.^8^ The laboratory baseline investigations required for ART initiation include screening for TB and cluster of differentiation 4 (CD4). The latter refers to a glycoprotein found on the surface of helper T-cells of the immune response. The CD4 count is used to assess progression of HIV disease, including risk for developing opportunistic infections and guide the use of preventive treatment.^8,9^ The CD4 count is used for reflexed cryptococcal disease screening for people living with HIV whose CD4 cell counts are < 200 cells/µL, as well as assessing eligibility for cotrimoxazole preventive therapy.^8^ In addition, renal sufficiency assessment, screening for anaemia, cervical cancer screening and assessing hepatitis B virus co-infection are required before the initiation of ART.^8^ These baseline laboratory investigations are requested by public sector health facilities and submitted to the local National Health Laboratory Service (NHLS) site.^10^

The NHLS is mandated to provide laboratory services for public sector health facilities.^10^ It has an extensive network that delivers laboratory and public health services to more than 80% of the population.^10^ Specimens from 3800 primary health care facilities and hospitals are collected, transported and delivered to these laboratories using an integrated hub-and-spoke courier system.^11,12^ Most laboratories offer a basic pathology repertoire, while specimens for specialised testing, such as CD4, HIV viral load and molecular TB, are referred within the tiered network.^13^ These services are distributed to maximise laboratory resources, including staff and appropriately placed infrastructure, which results in cost-saving and optimal turnaround time (TAT).^13^

Historically, CD4 testing was deployed at academic centres in South Africa based in urban centres.^14^ These were situated in the provinces of Gauteng, KwaZulu-Natal and the Western Cape. Two coverage analyses were conducted to assess equity of access for CD4 testing.^12,13^ Smith et al. conducted a locational analysis for the siting of HIV and AIDS diagnostic equipment using a modified set covering approach to develop a relational-algebraic capacitated location (RACL) algorithm that determined the optimal number of CD4 laboratories required to provide coverage within a defined travel time.^12^ Furthermore, any health facilities with poor access were deemed to require point-of-care testing (POCT).^12^ An estimated 61 laboratories and 20 POCT sites were identified to provide coverage, given a travel time of 2 h.^12^ Glencross et al. developed an integrated tiered service delivery model (ITSDM) that incorporated six service tiers to ensure accessible CD4 testing irrespective of geographic location as an extension of the then 2-tiered system in place (tiers 4 and 5).^13^ The main objective of this model was to identify and address coverage gaps. There were two POCT tiers proposed in this model: (1) remote and ‘hard-to-reach’ testing for a single ART facility and (2) a point-of-care (POC)-hub that would service up to 8–10 referring clinics.^13^ In contrast, laboratory tiers 3, 4 and 5 were assigned to accommodate the increasing service demands and higher workload volumes.^13^ The daily test volumes per tier are assigned as follows: (1) tier 1: < 10, (2) tier 2: < 30–40, (3) tier 3: < 150, (4) tier 4: < 300 and tier 5: > 600.^13^ Tier 6 is the supernumerary coordinating-umbrella responsible for coordinating, harmonisation and standardisation of testing, training and quality control across the national network of laboratories and related testing sites.^13^ This role is fulfilled by the National Priority Programmes of the NHLS.^10^ This study proposed that 21, 18 and 20 tier three (community), four (district) and five (tertiary) CD4 sites, respectively, be deployed to provide adequate coverage (*n* = 59 excluding tiers 1 and 2).^13^ Based on the coverage analysis, CD4 testing was implemented at most of the identified district and community laboratories, with some of the proposed sites not implemented because of operational challenges.^12,13,15,16,17^ Subsequent limited centralisation and decentralisation saw the number of testing facilities change over time, resulting in 47 CD4 testing laboratories across nine provinces from 2018 onwards.^15,16^

Local guidelines were developed to ensure optimal therapy and good clinical outcomes that included a decrease in HIV-related morbidity and mortality.^4^ The criteria for ART initiation in 2004 were a CD4 < 200 cells/µL or clinically staged as World Health Organization (WHO) III/IV (acquired immunodeficiency syndrome [AIDS]-defining illness).^1,4^ Local guidelines were changed to include all people living with HIV with a CD4 < 350 cells/µL from 2013.^18,19^ Antiretroviral therapy eligibility was further expanded by increasing the CD4 threshold to < 500 cells/µL in 2015, with universal test-and-treat (ART initiation regardless of CD4 count) introduced in 2016.^19,20,21^

The bulk of specimens are referred from non-CD4 testing NHLS laboratories closest to the referring health facilities. A local study reported that shorter interlaboratory referral distances resulted in lower laboratory-to-laboratory TAT.^22^ It is reasonable to assume that the introduction of lower service delivery tiers at district and community CD4 laboratories across South Africa would have reduced or maintained shorter interlaboratory referral distances.^15,17^

This study builds on the initial findings of a local analysis, which reported the impact of interlaboratory referral distances on TAT in 2018.^22^ This study expands on these initial findings by reporting changes in interlaboratory Euclidean distances (EDs) in the era of the tiered CD4 service model over an extended period, indicating the impact of centralisation or decentralisation of services on ED over time. This study aimed to assess the impact of implementing a tiered CD4 service model on interlaboratory referral distances between 2012 and 2021 in the public sector in South Africa.

## Research methods and design

### Study design

A retrospective study design was used to assess ED for the period between 2012 and 2021. Cluster of differentiation 4 testing was done using either the XL-MCL, FC500 MPL/CellMek or Aquios CL cytometers, supplied by Beckman Coulter (Miami, FL, United States [US]).^23^ All laboratories use standardised PanLeucoGating (PLG) reagents and standard operating procedures.^24^

### Setting

Data are reported for testing performed by NHLS, which has a mandate to provide laboratory services for public sector health facilities across South Africa.^10^ It has 268 laboratories across the nine provinces and serves approximately 80% of the South African population.^10^ Cluster of differentiation 4 testing was offered by NHLS laboratories that utilised a hub-and-spoke courier network to collect and transport specimens.

### Study population and sampling strategy

The study population consisted of all CD4 laboratories within the NHLS that offered testing during the study period, utilising convenience sampling. Data were only collected for tiers three, four and five as the NHLS has not implemented POCT (tiers 1 and 2).

### Data collection and preparation

The aggregate data extract was provided by the corporate data warehouse (CDW) as a password-protected file. It included the following variables: (1) year, (2) month, (3) source laboratory, (4) source laboratory latitude and longitude, (5) testing laboratory, (6) testing laboratory latitude and longitude, (7) province, (8) test volumes, (9) test volumes that met the TAT cut-off (40 h) and (10) test volumes that did not meet the TAT cut-off.^10^ The TAT cut-off in 2012 was set at 85% of resulting specimens within 48 h; but after 2016, the target time changed to 40 h, with an incremental increase in the expected percentage within the target to 95% by 2022. A referral was defined as the movement of specimens from source laboratories situated closest to health facilities.^11,12^ Specimens are registered using the laboratory information system (LIS) at the source laboratory closest to the health facility, with referrals to their designated specialised testing laboratory. Additional referrals may occur between testing laboratories in the case of equipment downtime or reagent outages (i.e., re-routing of tests). The CDW assigned the province based on the requesting health facility.

The ED was calculated in kilometres (km) for each pair of interlaboratory referrals (from the source to the testing site) using Microsoft Excel^®^ (Redmond, Washington, US). The ED formula was^25^:

$$ED=\sqrt{{\left(x2-x1\right)}^{2}{\left(y1-y1\right)}^{2}}.$$ [Eqn 1]

The latitudes and longitudes were used as *x* and *y*, respectively. The coordinates for the testing laboratory were *x*2 and *y*2, respectively (*x*1 and *y*1 were for the source laboratory). Two POWER formulae were used to calculate the differences between x and y values for each set of referrals, for example, –POWER(*x*2 – *x*1). The referral status was also assigned as a binary variable (1: different source and testing laboratory [referral] and 0: same source and testing laboratory [not a referral]). To calculate the ED, the SQRT formulae of the two calculated differences were determined. The calculated ED was categorised as: (1) ≤ 50 km, (2) 51 km – 99 km, (3) 100 km – 199 km, (4) 200 km – 299 km and (5) ≥ 300 km. The percentage of specimens that met the TAT cut-off was determined.

### Data analysis

The prepared data were analysed using Stata SE (College Station, Texas, US) and SAS 9.4 (Cary, North Carolina, US). Test volumes were assessed, with the percentage of referrals, median ED and interquartile ranges reported. The percentage of specimens with an ED category of ≤ 50 km, 51 km – 99 km, 100 km – 199 km, 200 km – 299 km and ≥ 300 km was determined. A scatter plot was used to visualise the relationship between referral volumes and ED (correlation coefficient results reported). The linear fit prediction plot was also added. Daily test volumes were extracted for each laboratory and assigned to tiers as follows: (1) < 150 km: tier 3, (2) 150 km – 300 km: tier 4 and (3) ≥ 300 km: tier 5. The tiers were assigned to each laboratory in the data extract. The percentage of specimens that met the TAT cut-off was categorised as 0% – 20%, 21% – 40%, 41% – 60%, 61% – 80% and 81% – 100%. The ED analysis was repeated at the provincial level. The provincial percentage change in the median ED and the percentage of referrals between 2012 and 2021 were determined. ArcGIS (ESRI, Redlands, Washington, US) was used to create a map to report the median ED by source laboratory for the 2021 calendar year, categorised into four buckets as follows: (1) 0 km – 100 km, (2) 101 km – 200 km, (3) 201 km – 300 km and (4) > 300 km. The map displayed the provinces using a shapefile obtained from the Municipal Demarcation Board of South Africa.^26^

### Ethical considerations

Ethical clearance to conduct this study was obtained from the University of the Witwatersrand Human Research Ethics Committee (Medical) (No. M220163).

Only aggregate secondary laboratory data were used; our study did not require the use of any patient identifiers or consent.

## Results

Data are reported for 30 821 314 specimens, of which 14 481 715 (47.0%) were referred. Euclidean distance reported a skewness of 2.23. Data are reported for 248 source laboratories, which referred specimens to 63 testing sites.

### National analysis

Annual test volumes decreased from 3 891 472 in 2014 to 2 225 674 in 2021 (Table 1). The number of CD4 testing laboratories decreased from 63 in 2012 to 47 in 2019, because of centralisation initiatives. Interlaboratory referrals contributed between 43.2% (2013) and 50.8% (2018). The median ED ranged from 55 (interquartile range [IQR]: 28–98) in 2012 to 60 km in 2018 (IQR: 27–99) and 2019 (IQR: 27–100). Overall, the median ED was 58 (IQR: 27–99). The percentage of specimens with an ED of ≤ 50 km decreased from 52.2% (2012) to 44.3% by 2019. In 2020 and 2021, this increased to 47.5% and 46.8%, respectively. Overall, the contributions per ED category of 51 km – 99 km, 100 km – 199 km, 200 km – 299 km and ≥ 300 km were 35.1%, 13.2%, 3.5% and 0.3%.

**TABLE 1:** Analysis of the annual percentage of referred cluster of differentiation 4 specimens and the median Euclidean distances in kilometres.

Year	Test volumes		CD4 testing laboratories (*n*)	Interlaboratory referrals		Euclidean distance (km)						
	*n*	%		*n*	%	Median	IQR	≤ 50	51–99	100–199	200–299	≥ 300
2012	2 775 449	9.0	63	1 257 344	45.3	55	28–98	52.2	30.9	11.4	5.2	0.3
2013	3 828 562	12.4	61	1 652 688	43.2	57	27–101	48.7	32.3	13.2	5.6	0.3
2014	3 891 472	12.6	61	1 714 480	44.1	57	27–101	48.6	34.1	14.4	2.7	0.2
2015	3 611 396	11.7	61	1 674 512	46.4	59	27–101	46.0	35.3	15.8	2.5	0.3
2016	3 394 412	11.0	52	1 676 935	49.4	57	27–98	48.0	35.4	13.9	2.4	0.3
2017	3 088 291	10.2	49	1 545 061	50.0	58	27–98	49.5	35.7	12.6	1.9	0.3
2018	2 829 404	9.2	47	1 437 897	50.8	60	27–99	47.5	36.2	13.1	3.0	0.2
2019	2 782 521	9.0	47	1 306 710	47.0	60	27–100	44.3	38.4	13.1	4.0	0.2
2020	2 394 133	7.8	47	1 146 366	47.9	58	25–98	47.5	36.6	11.7	4.0	0.2
2021	2 225 674	7.2	47	1 069 722	48.1	57	25–98	46.8	37.5	11.1	4.0	0.6

**Overall**	**30 821 314**	**100.0**	**-**	**14 481 715**	**47.0**	**58**	**27–99**	**47.9**	**35.1**	**13.2**	**3.5**	**0.3**

Note: The percentage of specimens with a Euclidean distance category of ≤ 50 km, 51 km – 99 km, 100 km – 199 km, 200 km – 299 km and ≥ 300 km was reported. Data report testing performed by the National Health Laboratory Service, which serves public sector health facilities in South Africa. The number of laboratories offering CD4 testing is also indicated.

CD4, Cluster of differentiation 4; IQR, interquartile range.

### Association between Euclidean distance, referral volumes, turnaround time performance and tier

**FIGURE 1:**
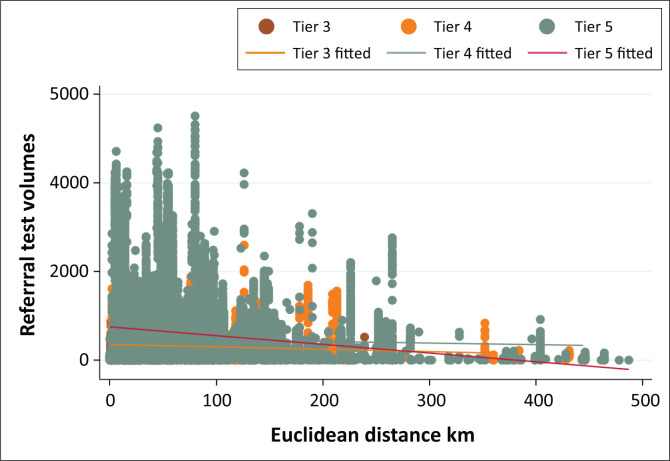
Scatter analysis of the relationship between cluster of differentiation 4 interlaboratory referrals, Euclidean distances in kilometres and referral volumes by laboratory tier between 2012 and 2021. Tiers 3, 4 and 5 were defined as daily volumes of < 150 km, 150 km – 300 km and ≥ 300 km. The linear fit prediction plot is also reported. The data are for tests performed by the National Health Laboratory Service, which serves public sector health facilities in South Africa.

**FIGURE 2:**
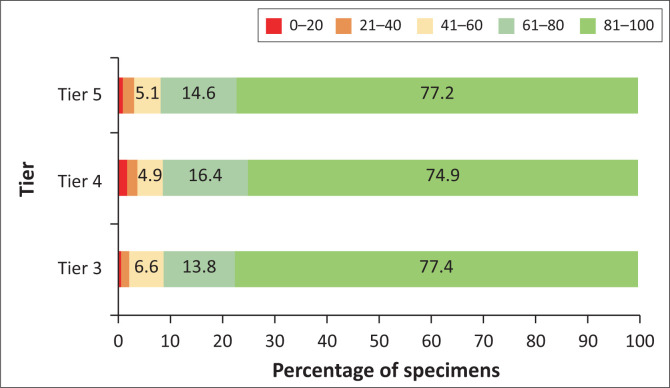
Analysis of the percentage of specimens that met the turnaround time cut-off of 40 h categorised as 0% – 20%, 21% – 40%, 41% – 60%, 61% – 80% and 81% – 100% reported by laboratory tier, which was defined as daily volumes of < 150 km, 150 km – 300 km and ≥ 300 km. Data are for tests performed between 2012 and 2021 by the National Health Laboratory Service, which serves public sector health facilities in South Africa.

The scatter plot of ED and referral volumes showed a significant negative linear correlation (–0.1540; *p* ≤ 0.001) (Figure 1). Most interlaboratory referrals appeared to be performed within an ED of 100 km, as confirmed by Table 1 (83.1%). Longer EDs were reported for tiers 4 and 5. Similar TAT performance was reported across three tiers (Figure 2). There were 77.4%, 74.9% and 77.2%, where 81% to 100% of specimens met the TAT cut-off for tiers 3, 4 and 5, respectively. Overall, 1.0%, 2.1%, 5.2%, 14.7% and 77.0% of specimens met the TAT cut-off at the categories 0% – 20%, 21% – 40%, 41% – 60%, 61% – 80% and 81% – 100%, respectively.

### Provincial analysis

The provincial analysis revealed that the percentage of referrals ranged from 22.8% (Northern Cape) to 69.1% (Limpopo) compared to the national value of 47.0% (Table 2). Most testing was performed in the KwaZulu-Natal (32.9%) and Gauteng (22.3%) provinces, where referrals comprised 48.6% and 43.3%. The median ED ranged from 16 (IQR: 8–45) (Gauteng) to 186 km (IQR: 186–206) (Northern Cape). A median ED below the national value of 58 km was also reported in the Free State (57 km, IQR: 7–161), KwaZulu-Natal (50 km, IQR: 20–80) and Western Cape (45 km, IQR: 16–108) provinces. The Eastern Cape, Limpopo and Mpumalanga provinces reported higher median ED values of 81 (IQR: 38–125), 81 (IQR: 42–111) and 83 (IQR: 56–91). With the exclusion of the Northern Cape province, the percentage of specimens with an ED of ≤ 100 km ranged from 65.5% (Eastern Cape) to 99.9% (Gauteng). An ED of ≥ 300 km was reported for 6.1% and 6.4% of specimens in the Northern Cape and Western Cape provinces.

**TABLE 2:** Provincial analysis of the test volumes, percentage of interlaboratory referrals and the median Euclidean distance in kilometres.

Province	Test volumes		Interlaboratory referrals		Overall Euclidean distance (km)						
	*n*	%	*n*	%	Median	IQR	≤ 50	51–99	100–199	200–299	≥ 300
Eastern Cape	3 288 936	10.7	1 829 101	55.6	81	38–125	31.5	34.0	27.5	7.0	0.0
Free State	1 614 658	5.2	729 477	45.2	57	7–161	9.6	59.8	8.8	21.7	0.2
Gauteng	6 887 583	22.3	2 983 452	43.3	16	8–45	68.6	31.3	0.0	0.0	0.0
KwaZulu-Natal	10 132 865	32.9	4 929 196	48.6	50	20–80	58.7	28.7	9.8	2.7	0.0
Limpopo	2 199 635	7.1	1 519 466	69.1	81	42–111	38.6	31.4	27.0	3.0	0.0
Mpumalanga	2 356 503	7.6	1 184 043	50.2	83	56–91	16.9	80.3	2.7	0.1	0.0
Northern Cape	544 758	1.8	127 010	23.3	186	186–206	0.0	0.0	84.5	9.4	6.1
North West	1 680 421	5.5	696 843	41.5	60	49–107	43.8	23.5	32.7	0.0	0.0
Western Cape	2 115 955	6.9	483 127	41.5	45	16–108	54.0	18.1	17.4	4.1	6.4

**Overall**	**30 821 314**	**100.0**	**14 481 715**	**47.0**	**58**	**27–99**	**47.9**	**35.1**	**13.2**	**3.5**	**0.3**

Note: The percentage of specimens with a Euclidean distance categorised as ≤ 50 km, 51 km – 99 km, 100 km – 199 km, 200 km – 299 km and ≥ 300 km was reported. Data report testing performed by the National Health Laboratory Service, which serves public sector health facilities in South Africa.

IQR, interquartile range.

### Provincial comparison: 2012 and 2021

At the national level, between 2012 and 2021, the median ED increased from 55 km to 57 km (3.6% change). The provincial percentage change in the median ED ranged from –55.7% (Free State) to 0.8% (Mpumalanga). The median ED also reported a percentage reduction of over 10% in the Limpopo and North West provinces (Table 3). In the KwaZulu-Natal province, the median ED increased from 44 km to 54 km (22.7% change). For the Free State province, a median ED decrease of 51 km was reported, with the percentage of referrals decreasing from 61.7% in 2012 to 41.6% by 2021. In contrast, for the KwaZulu-Natal province, the percentage of referrals increased from 35.4% to 57.1%. In the Northern Cape province, referrals reduced from 18.7% to 4.4% with a median ED decline of 20 km.

**TABLE 3:** Provincial analysis for the 2012 and 2021 calendar years to assess percentage change for the cluster of differentiation 4 interlaboratory referrals median Euclidean distance in kilometres.

Province	Median Euclidean distance			Percentage of referrals		
	2012	2021	% Change	2012	2021	% Change
Eastern Cape	78.0	81.0	3.8	47.8	60.5	12.6
Free State	115.0	51.0	−55.7	61.7	41.6	−20.1
Gauteng	16.0	15.5	−3.1	52.1	34.6	−17.5
KwaZulu-Natal	44.0	54.0	22.7	35.4	57.1	21.7
Limpopo	89.0	79.0	−11.2	69.3	74.4	5.1
Mpumalanga	63.5	64.0	0.8	51.4	51.6	0.2
Northern Cape	206.0	186.0	−9.7	18.7	4.4	−14.2
North West	60.0	54.0	−10.0	48.3	34.5	−13.8
Western Cape	43.0	45.0	4.7	21.5	25.2	3.6

**Overall**	**55.0**	**57.0**	**3.6**	**45.3**	**48.1**	**2.8**

Note: The percentage of specimens that were referred was also assessed, with the percentage change reported. Data report testing performed by the National Health Laboratory Service, which serves public sector health facilities in South Africa.

### Source laboratory median Euclidean distance analysis for 2021

**FIGURE 3:**
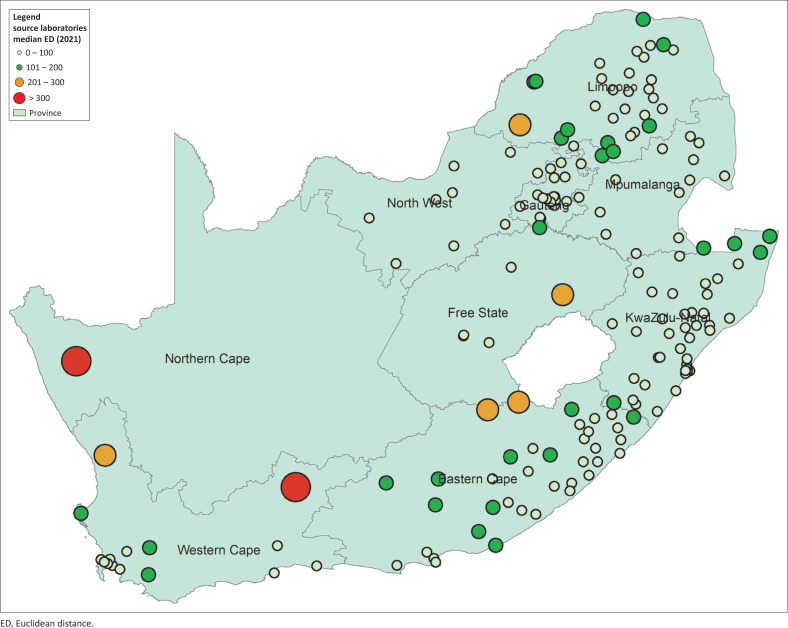
Analysis of the cluster of differentiation 4 interlaboratory referrals median Euclidean distances in kilometres for source laboratories in 2021 categorised into four buckets: (1) 0 km – 100 km, (2) 101 km – 200 km, (3) 201 km – 300 km and (4) > 300 km. Data report testing performed by the National Health Laboratory Service, which serves public sector health facilities in South Africa.

There were only two laboratories that reported a median ED > 300 km (Figure 3). Furthermore, there were five laboratories with a median ED of 200 km – 300 km. There were 172 out of 179 (96.1%) laboratories reported with a median ED < 200 km.

## Discussion

The study findings indicate that a tiered CD4 service that incorporated the majority of coverage analysis recommendations has resulted in a sustained median ED below 60 km at the national level and confirmed that less than half of the specimens tested were referred.^12,13^ The majority of referrals are performed in higher-tier laboratories in urban metropolitan areas, as confirmed by a national 75th percentile ED of < 100 km, further confirmed by 50% of referrals reporting an ED of under 50 km, which implies very short distances to testing facilities. It is also not surprising to find that referral volumes and TAT performance reported an inverse correlation with ED with outcomes reiterating the value of earlier coverage analysis conducted that improved access to CD4 services.^12,13^ There were very similar TAT performance findings reported by laboratory tier.

At the provincial level, there was a wide variation in the median ED. However, all nine provinces reported a median ED < 200 km, which is equivalent to a two (*n* = 2) hour travel time and confirms that the majority of the coverage gaps identified by the RACL and ITSDM models have been addressed at the provincial level.^12,13^ There was, however, a provincial variation in the percentage of specimens with an ED of ≤ 50 km and ≥ 200 km. A lower ED was predominantly reported for provinces that make up 62% of test volumes including Gauteng, KwaZulu-Natal and Western Cape. There was also a substantial variation in the percentage of referrals (samples referred to designated testing sites), with the Northern Cape being the lowest because of decentralisation of CD4 testing to the Upington, De Aar and Tshwaragano community laboratories (only one other laboratory does not offer CD4 testing).^15,17^ The Northern Cape province was the only province that reported a median ED exceeding 100 km despite the addition of community laboratories because of the vast travel distances between towns.^15,17^ This study confirms that services have been sustained since 2012 despite some decentralisation and consolidation of services.^15,16,17^ A local study reported that there were some over-serviced districts in the KwaZulu-Natal province, and sites were thus streamlined based on the ITSDM.^16^ The comparison of the median ED between 2012 and 2021 in this province confirmed the managed centralisation.^16^ Similarly, the decentralisation of CD4 services that took place in the Free State province resulted in the most significant reduction in the median ED. This demonstrates that the placement of CD4 testing laboratories is a combination of coverage analysis as well as operational conditions within the laboratory, that in some instances, require consolidation or decentralisation.^12,13^

The 2021 analysis of source laboratory median ED showed that 7 out of 179 (3.9%) laboratories exceeded the 200 km radius for referrals recommended by earlier coverage analysis.^12,13^ However, only two laboratories reported a median ED over 30 km. Both these laboratories, namely Springbok and Beaufort West, were proposed in the coverage analysis but were never implemented because of operational challenges.^12,13^ These are small clinical pathology laboratories in rural areas with low population density, often operated by a single medical technologist. Laboratories such as Upington, De Aar and Tshwaragano faced similar challenges and successfully integrated CD4 testing.^15,17^ These laboratories managed to introduce CD4 testing to the introduction of the walk-away, fully automated Aquios CL cytometers, which use a load-and-go system that combines primary specimen preparation and analysis.^27^ With this new platform, CD4 testing could potentially be offered at the community Springbok and Beaufort West laboratories, which would improve equity of access and reduce ED.

The findings also show that when compared to the use of more complicated specimen referral networks, using ED can identify coverage gaps and enable optimal access to services.^28^ However, for countries with well-established diagnostic networks that have conducted coverage analysis, ED could provide the same findings with far less effort and cost.^12,13^

Subsequent to 2011, because of multiple guideline changes, the percentage change in annual test volumes ranged from –13.4% (2020 vs. 2019) to 2.8% (2014 vs. 2013).^9,29,30,31,32,33^ These included the 2016 introduction of universal test-and-treat guidelines, which permitted ART initiation without a confirmed CD4 count.^29^ This resulted in a reduction in CD4 volumes ranging between 6.0% and 9.0% for calendar years 2015 to 2018 (compared to previous years).

This study emphasises the value of laboratory data that is made possible by a single national laboratory information database repository.^34^ The use of these data supports the extraction of important insights into the logistics and related strengths and weaknesses of laboratory operations, as demonstrated by the combination of centralised tier five services matched with decentralised community laboratories that improve coverage.^13^ This approach could be applied to other specialised testing, such as testing for chronic diseases and cancer screening, where referrals are required. Future ED analysis, on specialised testing, could result in service improvements, which could positively impact health care outcomes.

## Conclusion

The study findings indicate that a tiered CD4 service addressing coverage gaps resulted in a sustained national median ED below 60 km between 2012 and 2021 across the public sector diagnostic services in South Africa. These findings demonstrate that in more mature laboratory services, the ED could provide similar results for diagnostic network optimisation analysis.

### Limitations

A limitation of using ED is that it may have underestimated travel times. This may be more predominant in rural settings, where geographical features result in longer travel times. This study did not assess the distance from the health facility to the source laboratory, which may have identified additional coverage gaps. These findings are consistent with earlier coverage analyses that used facility data.^12^ Additional analysis could include geospatial hotspot assessment.^35^ The current reported analysis was only conducted for public sector laboratories in South Africa.
